# The Creation of a Competent Global Regulatory Workforce

**DOI:** 10.3389/fphar.2019.00181

**Published:** 2019-03-19

**Authors:** William Bridges

**Affiliations:** Regulatory Affairs Professionals Society, Rockville, MD, United States

**Keywords:** competency, competency model, entrustable professional activity, behavioral indicator, regulatory affairs, regulatory professional

## Abstract

Regulatory affairs professionals play pivotal roles in ensuring healthcare products adhere to regulations and in gaining regulatory approval for product manufacture and sales. To do this, they must understand the science and technology connected with a product, the company's business goals, and, most importantly, the nuances of national and international regulations and guidances connected to the product. But although they perform complicated work connected to the entire product development lifecycle, surveys have indicated only 14% of regulatory professionals come to the field with a degree related to the work and for more than half, regulatory work is a “second career.” The net result is a heterogeneous professional population that must learn complex, detailed work on the fly in as short a time as possible. Without a structure to guide development, these expectations are a challenge for someone new to the field, that person's supervisor, and for training developers. Various non-profit groups have created competency models to provide this structure, but because competencies only identify traits demonstrated by high-performing professionals, not the specific tasks associated with individual roles, these models have had limited impact on the profession. Identifying and structuring actionable tasks based on a competency model would increase the model's utility, dissemination, and usage. Entrustable professional activities might provide the methodology for doing so.

## Introduction

Regulatory professionals working for healthcare product companies impact nearly all phases of a healthcare product's lifecycle as part of their work to gain and maintain regulatory approval. But although they share the common goal of ensuring product safety through regulatory compliance, incredible variations exist in the scope and responsibility of a regulatory professional's work, depending on company size, organization, product portfolio, and development timeline. Thus, a single regulatory professional may do any combination of the following:

- Contribute to creating the regulatory strategy related to a product's eventual approval, which requires a strong command of international regulatory authorities' regulations and guidances.
- Collaborate with product developmental teams, from working with engineers on a medical device in the design phase to researchers during the clinical phase to advertising teams in the promotion and labeling phase.
- Ensure adherence to manufacturing requirements, which can include guiding inspections of manufacturing facilities and coordinating the company's response to inspection results.
- Gather all materials generated in the research and development, preclinical or non-clinical, and clinical phases to create a submission packet for regulatory approval, then shepherding the application throughout the approval process.
- Monitor product for any post-approval changes that may impact the original approval parameters.
- Comply with requirements for periodic and annual reports and adverse event reporting.

Their participation is pivotal to a product ever receiving regulatory approval, so they must be involved in every stage, providing strategic, tactical, and operational direction and support for working within regulations to expedite a product's development and delivery (Regulatory Affairs Professionals Society, 2015). Despite this important role, few regulatory professionals come into the field directly from an undergraduate program and most come from a career in another field or profession.

Every 2 years, the Regulatory Affairs Professionals Society (RAPS) surveys regulatory professionals around the globe about their work settings, educational backgrounds, compensation levels, and other demographic and work information. In the 2018 study, 86% of 2305 respondents reported they had degrees in something besides regulatory affairs, and most respondents indicated they came to regulatory affairs after working in another profession, including quality assurance/quality control (18% of respondents), research and development (12%), life sciences research (7%), engineering (7%), and clinical research (5%) (Regulatory Affairs Professionals Society, 2018). The disparate backgrounds of regulatory professionals and their complex, varied roles in product development make it challenging to provide a “one size fits all” training and development solution. Competencies and competency models can provide a structure for their professional development by highlighting the “unique characteristics of the most successful or even outstanding” regulatory professionals, although using competencies to create development plans can be challenging (Graber and Rothwell, 2010).

## Regulatory Competency Models

Many groups have created competency models for regulatory professionals, including The Organization for Professional in Regulatory Affairs, or (The Organization for Professionals in Regulatory Affairs (TOPRA), 2018), the Association of Graduate Regulatory Educators, or (Association for Graduate Regulatory Educators (AGRE), 2014), and RAPS in 2013, and 2015. (Refer to Table 1 for a comparison of regulatory professional competency models). RAPS's experience with developing and disseminating their frameworks highlights competency model potential benefits, but also shows their limitations and hints at ways in which their utility may be improved.

**Table 1:** Comparing domain topics across five regulatory professional competency models.

**Domain topic area**	**Professional development framework (RAPS, 2013)**	**Core competencies for graduates of MS programs in regulatory studies (AGRE, 2014)**	**Regulatory competency framework (RAPS, 2015)**	**Regulatory competency framework, updated (RAPS, 2018–2019)**	**Regulatory affairs competency framework (TOPRA, 2018)**
General, foundational information	Knowledge, Skills, and Abilities Throughout the Product Lifecycle		Scientific and health concepts	Regulatory strategy and planning	Strategy and technical
Strategic planning	Strategic planning	Strategy	Regulatory frameworks and strategy	Regulatory strategy and planning	Strategy
Premarketing/preapproval	Premarketing	Regulations, clinical, quality	Product development and registration	Premarketing/Post-marketing	Technical
Post-marketing/post-approval	Post-marketing	Regulations	Post-approval/Post-market	Premarketing/Post-marketing	Technical
Communication and soft skills	Interfacing	Communication	Communication	Professional development	Communication
Leadership			Leadership	Management and leadership	Core
Business acumen	Strategy	Strategy	Business acumen	Professional development	Business and Organizational Awareness
Ethics			Ethics	Professional development	Core

## The Regulatory Affairs Professional Development Framework

In 1990, RAPS created the Regulatory Affairs Certification (RAC), based on job analyses of regulatory professionals with 3–5 years of experience. But although a solid step toward identifying the knowledge and skills of a competent regulatory professional, it did not fulfill the need for a “true” competency model because of its focus on a specific portion of a regulatory professional's career and on product lifecycle-related items.

Recognizing that the profession needed a more complete competency picture, in 2003 RAPS initiated work on developing a full competency model that would create a more holistic vision of the successful regulatory professional and identify how those competencies morphed through various stages in a professional's career. From 2003 to 2007, senior regulatory professionals representing different product sectors, professional positions, company structures, and geographic responsibilities created comprehensive outlines of a regulatory professional's practice and associated knowledge and skills at different career stages. Developers then validated those outlines through comprehensive surveys followed by a series of focus groups. By the end of the process, more than 500 regulatory professionals had participated in the development and validation processes over a 2-year period to create the Regulatory Affairs Professional Development Framework (PD Framework).

An early decision in development was that the PD Framework should be as universal as possible, which meant it would be:

- Role-agnostic, so it should be applicable for regulatory professionals in industry, government, research, clinical, and other settings.
- Product-agnostic, so it would not mention sector-specific regulatory processes.
- Region-agnostic, so it would not mention specific regulatory authorities or guidances.
- Role-agnostic, so it would not provide details related to a regulatory professional's specific knowledge, skills, or competencies.

PD Framework developers matrixed competencies along two dimensions: level and domain.

Level referred to four stages of a regulatory professional's career:

- Level I: New to the field. Comes to the position with professional skills, such as basic project management and communications, so must focus on learning regulatory frameworks, requirements, legislation, and processes.
- Level II: Builds on that foundation and by the end of Level II, should be familiar with all regulatory tasks connected with his or her company's product lifecycle and submission process. These expectations mirrored the items in the RAC exam outline's topics, so the logical expectation was that the professional should earn the RAC by the end of this level after roughly 5 years of experience.
- Level III: Transitions from working entirely at the tactical level into a role that leverages technical knowledge into strategy. Often, the professional also becomes a manager of lower level regulatory professionals.
- Level IV: Shifts almost completely out of direct tactical regulatory work to be strategic regulatory lead, which includes developing new approaches for achieving or defining business objectives that build on his or her strong understanding of regulatory requirements, opportunities, risks, and alternatives.

Domains were logical subdivisions within the professional's scope of responsibilities:

- Strategic planning, which included regulatory strategy-related work throughout the lifecycle, organization of regulatory information and knowledge, integration of regulatory perspectives into the organization, and regulatory policies and procedures.
- Premarketing, which included any regulatory work connected to the research and development, preclinical, and clinical phases through submission/registration.
- Post-marketing, which involved reporting, compliance, and post-market surveillance, as well as labeling, advertising, and promotion.
- Interfacing responsibilities extended throughout the lifecycle and included communication and interaction within the organization, with regulatory agencies, professional trade, standards organizations, and with other stakeholders.

Although developers didn't want the PD Framework model to provide granular details about a regulatory professional's work, they did include overviews of the knowledge, skills and abilities of the regulatory professional at each level by domain (Regulatory Affairs Professionals Society, 2007).

## Developing the Regulatory Competency Framework

By their nature, competency models identify the characteristics of excellent performance at a specific moment of time. As such, organizations must revisit them periodically to ensure accuracy (Graber and Rothwell, 2010). In 2015, RAPS staff and 15–20 subject matter experts, did this with the PD Framework and created the Regulatory Competency Framework (RCF). Supplementary Material: (Regulatory Affairs Professionals Society, 2015).

The largest difference between PD Framework and RCF was expansion from four domains to eight, in the hope that they would better represent both regulatory-specific and general professional competencies:

- Scientific and Health Concepts: Understanding and application of evolving basic and translational science, regulatory science and public health concepts to drive new approaches to improve the development, review, and oversight of healthcare products.
- Ethics: Ability to integrate and demonstrate core values, integrity, and accountability.
- Business Acumen: Ability to leverage systems and processes to successfully operate a regulatory function.
- Communication: Ability to clearly convey or exchange information with stakeholders within and outside the organization.
- Leadership: Ability to direct and contribute to initiatives within the organization, with groups engaged in developing good regulatory practice and policy, and within the regulatory profession. Ability to provide clarity and direction amid complexity and develop solutions for self, colleagues and the organization.
- Regulatory Frameworks and Strategy: Knowledge of regulatory frameworks and external environments and the ability to apply these to regulatory solutions throughout the product lifecycle.
- Product Development and Registration: Knowledge of the research and development, preclinical and clinical steps and related regulations in healthcare product development.
- Post-approval/Post-market: Knowledge of requirements and processes for maintaining a product on the market, reporting and surveillance (Regulatory Affairs Professionals Society, 2015).

## Updating the RCF

Although the RCF provided a solid competency model, data gathered in 2018 suggested challenges in its application. Based on surveys and education-related evaluations, over half of RAPS 16,000 members were aware of the RCF, but fewer than 100 individuals had downloaded the RCF from its 2015 release until late 2017. Furthermore, only a handful of companies had followed the RCF's recommendations of tailoring its general competencies to their specific products and organization. Instead, they used it to create position descriptions, which demonstrated a complete misunderstanding of how to use competency models (Graber and Rothwell, 2010). When asked why, respondents said consistently that the model “lacked real world applicability” because of its high-level overview and sector-agnostic approach.

The information led to a reassessment of the RCF. Reviewers agreed that the RCF needed improvements, like removing redundancies and addressing gaps in professional skill competencies, but decided to maintain the RCF's high-level, universal view of regulatory competencies. However, in recognition of comments about lack of applicability, they created additional tools to help individuals use the model.

Improvements to the RCF's content included the following:

- The Ethics domain both contained competencies better suited to other domains and failed to capture the ethics of regulatory work as elegantly as the preexisting RAPS Code of Ethics. Supplementary Material: RAPS Code of Ethics. As such, the updated RCF guided regulatory professionals to become familiar with that document and included only a few ethics-related competencies.
- Management competencies, such as developing subordinates and identifying team resource needs, added to existing competencies to create a Management and Leadership domain.
- Developers condensed the premarketing and post-marketing domains into one.
- They eliminated redundancies.
- Items from the Scientific and Health Concepts domain moved to Regulatory Strategy and Planning.
- The subject matter experts created a Professional Development domain to hold competencies that all professionals should develop, including items from the Business Acumen and Communication domains.

The greatest alteration in the updated RCF was that it would be not one, but three, tools:

- The RCF remained a high-level, universal vision of competencies evidenced by the most successful regulatory professional at all four career levels. Unlike the previous version, however, the update included more guidance on how to apply the competencies to an individual. The hope is that for many, the additional assistance will be sufficient.
- However, developers recognized that many people will need help interpreting the RCF's intentionally-broad competencies, so they created *behavioral indicators* to provide examples of behaviors that lead to achievement of the RCF competency. For example, a competency in the Regulatory Strategy and Planning domain is “Participates in SOP development and training related to them.” Behavioral indicators for this competency include “Identifies the need for new regulatory procedures and SOPs and participates in development and implementation, helps train stakeholders on current and new regulatory requirements to ensure organization-wide compliance, and assists other departments in the development of SOPs to ensure regulatory compliance.” Developers will emphasize that the behavioral indicators cannot possibly encompass all the variations that exist from setting to setting and individuals should exercise judgment in which ones apply (Graber and Rothwell, 2010).
- For some, though, the competency model's inherent emphasis on behaviors makes it too difficult to see its application to his or her work tasks, and it is here that entrustable professional activities (EPAs) offer a solution.

EPAs first became prevalent in competency-based medical education because educators worried that although competencies excelled at describing a high-performing doctor's attributes, they did not describe the tasks that doctor should be expected to do. An EPA defines a discrete, easily measured unit of work. Thus, while competencies define a person, EPAs define that person's work.

**Figure 1:**
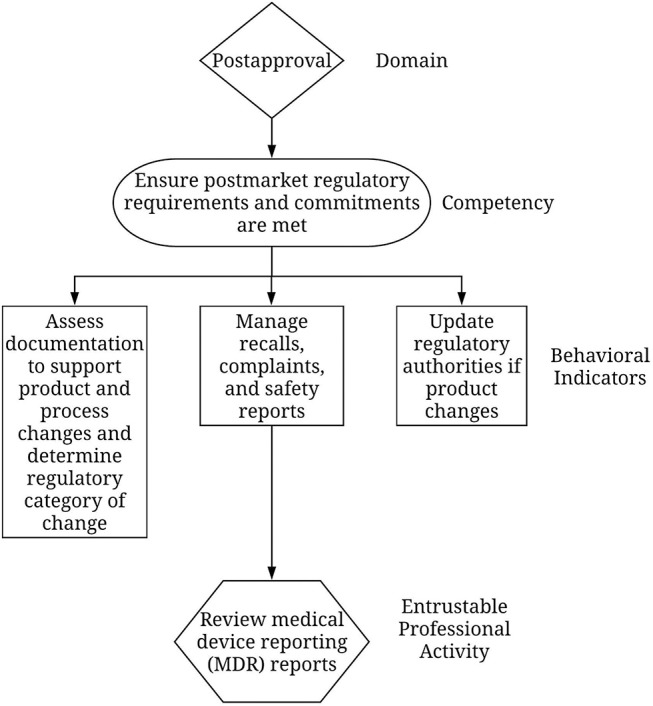
Example of competency-behavioral indicator-EPA relationship.

For regulatory professionals, developers took the competencies, established what work outcomes should come from each, then created lists of sector-specific EPAs that, when taken together, described most of the work connected with that competency. Developers then grouped EPAs into logical clusters to make it easier for someone using the structure to readily identify overarching areas for development. Supplementary Material: 2018 Update to the RCF. Figure 1 provides an example.

## Advantages of the updated RCF and the future of competency-based training for regulatory professionals

Developers expect the updated RCF will have a huge impact on the profession in multiple ways:

1. The multi-dimensional approach offers more flexibility and support, so those who are comfortable using competency models can use the RCF. Meanwhile, those who are new to the profession or may be the sole regulatory professional in his or her company will have the EPAs to highlight some of the tasks expected of regulatory professionals, as well as create clear metrics of ability related to career level.
2. The renewed emphasis on the RAPS Code of Ethics will underscore the vital role that regulatory professionals have in providing new, *safe* treatments for patients.
3. The additions of the professional, management, and leadership skills will create more well-rounded professionals who will be able to contribute more to their organizations' growth.
4. The more complete competency picture begins to create a better career “roadmap” for a profession that until this time has lacked a structural picture.
5. It also provides those who create training or other products to map their content against both behaviors and EPAs, which will help the individual achieve the level of mastery expected of the professional.
6. Related to the prior point, the updated model paves the way for a shift to competency-based training.

Traditional training development follows the ADDIE method, an acronym for Analysis, Design, Develop, Implement, and Evaluate. Analysis focuses on assessing needs, often for a large group of professionals, related to performing a specific task. Developers create content based on those needs with the assumption that all learners need the same information. The shift to competency-based education changes that process in some important ways.

Because competencies focus on high-performing individuals rather than what knowledge, skills, or tasks a specific role needs, analysis must shift to determining gaps that exist between a specific individual's performance level and the idealized competency. The result will be content tailored for the individual, rather than content that treats all workers as having the same level of need.

Because of this shift to understanding how the individual compares to the high performer, competency-based training will put more pressure on the individual to work proactively to identify gaps in his or her performance and seek training that addresses those gaps. It will also demand more communication between learner and supervisor, both to identify needs and to create mutually-acceptable ways to measure when or if the learner has filled those gaps.

The shift to competency-based training will be a slow process and will involve more work on the part of trainers, supervisors, and the individual regulatory professional. However, the connection to competencies will also result in a more well-rounded professional who will be fully conversant in the tasks in the regulatory lifecycle and in the communication, business, and leadership skills expected of all twenty first century healthcare professionals.

## Author Contributions

WB performed the research and wrote the manuscript. All Supplementary Materials created by the Regulatory Affairs Professionals Society.

### Conflict of Interest Statement

The author declares that the research was conducted in the absence of any commercial or financial relationships that could be construed as a potential conflict of interest. The handling editor HS and reviewers SK-F and PDS declared their involvement as co-editors in the Research Topic, and confirm the absence of any other collaboration.
