# Supplementary material for: The Creation of a Competent Global Regulatory Workforce
Source: Front Pharmacol. 2019 Mar 19;10:181. doi: 10.3389/fphar.2019.00181 (PMC6433988; doi:10.3389/fphar.2019.00181)
Supplement: Supplementary file 1 [file Data_Sheet_1.PDF]

RAPS REGULATORY AFFAIRS PROFESSIONALS SOCIETY
Driving Regulatory Excellence™

REGULATORY COMPETENCY FRAMEWORK AND GUIDE

RAPS.org

CONTENTS

Introduction 3

Overview of the Regulatory Competency Framework 3

Use of the Framework 5

Regulatory Competency Framework 6

INTRODUCTION

Regulatory professionals play critical roles throughout the healthcare product lifecycle, from concept through product obsolescence. They provide strategic, tactical and operational direction and support for working within regulations to expedite the development and delivery of safe and effective products around the world. Continuous evolution in science and changes in the regulatory environment, health sector and general economics each shape the dynamic and expanding scope of the regulatory profession.

Like all professions, regulatory is based on a shared set of competencies. The following Regulatory Competency Framework (RCF) from the Regulatory Affairs Professionals Society (RAPS) describes the essential elements of what is required of regulatory professionals at four major career and professional levels. The Framework breaks down the profession's basic competencies into clear, easy-to-understand statements and provides a foundation for regulatory professionals, regulators and employers to design training, develop curriculum, and manage professional and career development.

This framework is the result of years of research and development carried out by RAPS with the involvement of members and volunteer experts worldwide. Creating the Framework would not have been possible without the commitment and hard work of these dedicated individuals.

Care was taken to ensure the Framework applies to professionals in industry, government, research, and clinical and other settings—and is "product-agnostic." The Framework is designed to be globally relevant and is not based on any specific regulatory systems or geographic regions. It is not intended to provide all the details of the scope of practice or responsibilities of all regulatory professionals. Nor is it intended to be a prescriptive description of the required knowledge, skills or competencies of the professional. It is a basic framework that may be applied to many different circumstances. It is intended to be customized, built upon and used to create more detailed profiles for specific situations.

OVERVIEW OF THE REGULATORY COMPETENCY FRAMEWORK

The Framework is based on two primary dimensions: levels and domains. Levels refer to four distinct professional levels, while domains reflect broad categories of professional responsibilities. For each level and domain, the Framework presents statements that describe what a regulatory professional is expected to do. These statements can be used as a foundation for developing organization-specific competency models.

Professional Levels

While the Framework itself does not assign specific job titles to each level, examples of relevant titles are included in the summaries below.

Level I
Professionals at this level acquire knowledge related to the regulation of healthcare products, including regulatory frameworks, requirements, legislation and processes. Level I professionals should possess skills such as basic project management, communications, interpersonal skills and the ability to understand scientific and health concepts. Typical job titles at this level are coordinator, specialist and associate in some settings.

Level II
Professionals at this level have a strong foundation in the regulatory profession, including scientific, legal, policy and regulatory process management. They have well-developed regulatory technical knowledge and skills. Regulatory Affairs Certification (RAC) is targeted to professionals at this level. Typical job titles at this level are manager, senior manager in private sector; reviewer in regulatory agencies.

Level III
Professionals at this level understand and translate regulatory, scientific, operational and business knowledge into effective implementation plans and strategy. This level represents the successful transition from technical and tactical regulatory expertise to a role that integrates technical knowledge with management and strategy. Typical job titles at this level are director in private sector; senior or experienced reviewer, section managers in regulatory agencies.

Level IV
Professionals at this level take on the role of the strategic regulatory lead while developing new approaches for achieving or defining business objectives. Strategic planning and working with other teams throughout the product lifecycle—both within and outside the individual's organization—are among the most important responsibilities. These professionals must be able to navigate ambiguity, and demonstrate agility and other executive characteristics. They must possess and communicate strong understanding of the requirements, opportunities, risks and alternatives for developing and maintaining products. Typical job titles at this level are vice president, executive director, CEO in the private sector; division director in regulatory agencies.

Domains

Domains represent the major content categories across all levels of the Framework. Each of the eight domains reflects a major area of tasks and responsibilities of the regulatory professional:

- **Scientific and Health Concepts:** Understanding and application of evolving basic and translational science, regulatory science and public health concepts to drive new approaches to improve the development, review and oversight of healthcare products
- **Ethics:** Ability to integrate and demonstrate core values, integrity and accountability
- **Business Acumen:** Ability to leverage systems and processes to successfully operate a regulatory function
- **Communication:** Ability to clearly convey or exchange information with stakeholders within and outside the organization
- **Leadership:** Ability to direct and contribute to initiatives within the organization, with groups engaged in developing good regulatory practice and policy, and within the regulatory profession. Ability to provide clarity and direction amid complexity and develop solutions for self, colleagues and the organization
- **Regulatory Frameworks and Strategy:** Knowledge of regulatory frameworks and external environments and the ability to apply these to regulatory solutions throughout the product lifecycle
- **Product Development and Registration:** Knowledge of the research and development, preclinical and clinical steps and related regulations in healthcare product development
- **Postapproval/Postmarket:** Knowledge of requirements and processes for maintaining a product on the market, reporting and surveillance

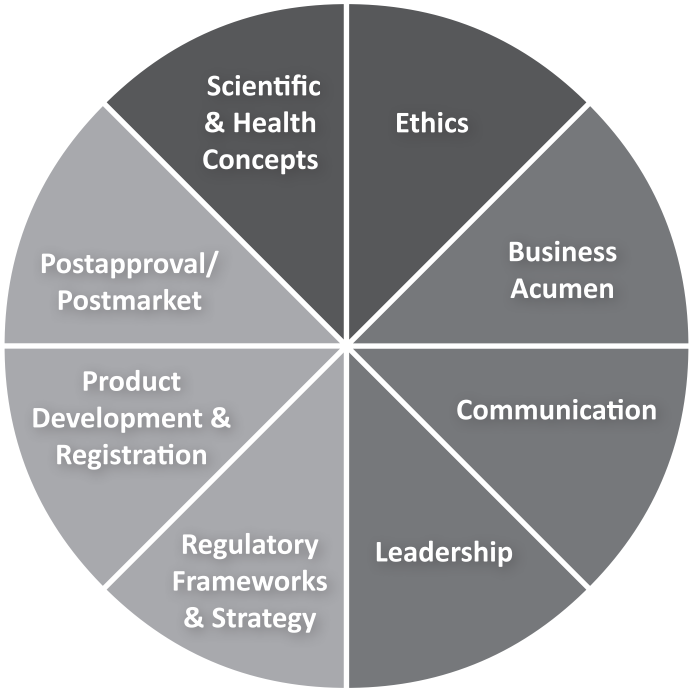

Regulatory Competency Domains – Across the eight domains, the product lifecycle, good science and ethical practices combine with core interpersonal competencies to drive performance excellence.

The Framework summarizes what a regulatory professional is expected to do at each level and within each domain. Many elements will apply to all professional levels. Responsibilities and tasks listed under each domain represent the major activities reported by regulatory professionals at each level. Generally, during early professional stages (levels I and II), the regulatory professional builds a solid understanding of regulations, regulatory processes and procedures. The application of this knowledge and the skills at these levels are typically tactical. At levels III and IV, regulatory professionals build on their knowledge and skill base, adding increasing strategic and management qualifications. Professionals at these levels often are less focused on tactical elements of regulatory affairs and more engaged in broad-scale, strategic responsibilities.

It is important to note that some responsibilities and tasks apply to multiple phases in the product lifecycle, even if they are listed under one domain. Further, the responsibilities and tasks described may not fully capture the activities of each regulatory professional. The nature of the organization, the educational background and previous experience of the professional and other factors may call for greater or lesser emphasis on certain responsibilities listed in the Framework or warrant additional tasks or responsibilities.

USE OF THE FRAMEWORK

The Regulatory Competency Framework provides an outline of the work performed by regulatory professionals and serves as the foundation for building tools to help individual professionals in planning and managing their career and professional development, and to assist regulatory agencies and regulatory employers with managing, coaching and mentoring regulatory talent. The Framework is not intended to be detailed or precisely tailored to the specific structure, product focus, or nature of every organization. More work will need to be undertaken to build out details that reflect the needs of individuals within specific environments. Some suggestions on how to use the Framework are described below.

Regulatory Professionals

Regulatory professionals can use the Framework as a checklist to improve performance in a current role. It is useful for building and executing a professional development plan (generally over a 12-month period) to strengthen individual competencies in target areas. The target areas chosen for emphasis should be based upon a comparison of your skill profile against the competencies necessary to accomplish specific needs or to perform a specific job function that is part of your current role. Additional input from your manager or others will be needed as you finalize the plan.

To build a career development plan to help you prepare for a new role, you should use the level of the target positon in the Framework to assess what additional skills are needed to effectively perform at the new job level. Career plans generally take 18 months or longer to fully execute. The Framework can serve as a foundational checklist that helps you define where your gaps in competencies are. Again, additional input from others may be helpful.

Regulators And Employers

Organizations can use the Framework as a starting place for processes that ultimately result in:

- Competency models for specific departments and job categories
- Curriculum designed for regulatory professionals at a variety of levels
- Instructional design strategies based upon employee results required by level
- Departmental and organization-wide training plans
- Department or organization-wide development and career planning efforts
- Foundational career paths for regulatory professionals
- Position descriptions and job postings
- Early stage succession planning and talent management

Additional tools and approaches to customize and refine the Framework are available from RAPS.

For more information about the Framework and career development tools for regulatory professionals, visit **RAPS.org**. To learn more about using the Framework for your organization, contact enterprise@raps.org.

LEVEL I

Level 1 professionals acquire knowledge related to the regulation of healthcare products including regulatory frameworks, requirements, legislation, and processes. Level I professionals should possess skills such as basic project management, communication and interpersonal skills, and an ability to understand scientific and health concepts. (Typical job titles at this level: coordinator, specialist, associate in some settings)

Level I
**Regulatory Frameworks & Strategy:** Knowledge of regulatory frameworks and external environments, and the ability to apply these to regulatory solutions throughout the product lifecycle.
• Identifies information sources and resources for local, regional and global regulations.<br>• Collects, organizes and maintains files on local, regional and global regulatory intelligence and other related information.<br>• Monitors the regulatory environment (specific regulations, guidance and other relevant information by product types, geography, etc.) and maintains information resources.<br>• Provides information used to evaluate proposed products for regulatory classification and jurisdiction.<br>• Researches requirements (local, national, international) and options for regulatory submissions, approval pathways and compliance activities.<br>• Assists in the development of regulatory procedures and SOPs.
**Product Development & Registration:** Knowledge of the research and development, preclinical and clinical steps, and related regulations in healthcare product development.
• Collects and organizes information on regulatory requirements for quality, preclinical and clinical data to meet applicable regulations.<br>• Organizes materials from preclinical and clinical studies for review and assists in the review process.<br>• Compiles and organizes materials for pre-submission reports and communications.<br>• Assists in the preparation of dossiers and submission packages for regulatory agencies.<br>• Tracks the status of applications under regulatory review and provides updates to the regulatory team.<br>• Maintains logs of communication and outcomes with regulators and other relevant internal or external stakeholders.<br>• Assists in the scheduling of meetings with internal stakeholders and regulators and develops and organizes materials for these meetings.
**Postapproval/Postmarket:** Knowledge of requirements and processes to maintain a product on the market; reporting and surveillance.
• Maintains systems to trigger and log regulatory reporting.<br>• Assists in the preparation and sending of postmarket reports and submissions.<br>• Tracks and maintains files on annual licenses, registrations and listings.<br>• Maintains systems to track, manage and report product-associated events.<br>• Tracks product complaints, events and recalls.
**Scientific & Health Concepts:** Understanding and application of evolving basic and translational science, regulatory science and public health to drive new approaches to improve the development, review and oversight of healthcare products.
• Understands scientific and health principles related to healthcare product development and regulations.<br>• Tracks scientific and/or clinical advances that impact healthcare product development and regulations.
**Ethics:** Ability to integrate and demonstrate core values, integrity and accountability throughout the organization and externally.
• Demonstrates ethical behavior by ensuring integrity in personal and organizational practices; respects people and principles, including professional, ethical and human values.<br>• Accountable for own behavior and actions.<br>• Abides by and upholds the laws and regulations of the authorities under which he or she operates and the organization's internal/external policies and directives.<br>• Demonstrates the importance of working together in the spirit of openness, honesty and transparency that encourages engagement, collaboration, respectful interactions and trust.<br>• Takes all possible steps to prevent and resolve any real, apparent or potential conflicts of interest between work responsibilities and private affairs.<br>• Raises and escalates, as appropriate, significant organizational ethics and compliance issues.
**Communication:** Ability to clearly convey or exchange information with stakeholders within and outside the organization.
• Clearly conveys information to peers, supervisors and other stakeholders.<br>• Assists in preparation for meetings with regulatory agencies and other stakeholders.<br>• Assists in the preparation of briefings and other information documents.<br>• Communicates information on regulatory requirements to other departments and business units.<br>• Composes routine communications with regulators and other key stakeholders.

Level I
**Business Acumen:** Leverages systems and processes to successfully operate a regulatory function.
• Preserves confidentiality of product information as appropriate.<br>• Maintains information systems (electronic and paper) for regulatory information and reports.<br>• Tracks the staging of activities and milestones in regulatory work plans.<br>• Develops/expands project management capabilities.
**Leadership:** Ability to direct and contribute to initiatives within the organization, with groups engaged in the development of good regulatory practice and policy, and within the regulatory profession.
• Creates clarity and direction amid complexity and develops solutions for self, colleagues and the organization.<br>• Seeks out diverse ideas, opinions and insights, and applies them in workplace.<br>• Connects and relates well with people who think and act differently than oneself.<br>• Embraces scrutiny and accepts feedback as opportunity to learn and improve.

LEVEL II

Level II professionals have a strong foundation in the regulatory profession, including scientific, legal, policy and regulatory process management. They have well developed regulatory technical knowledge and skills. (Typical job titles at this level: manager, senior manager in private sector; reviewer in regulatory agencies)

Level II
**Regulatory Frameworks & Strategy:** Knowledge of regulatory frameworks and external environments, and the ability to apply these to regulatory solutions throughout the product lifecycle.
• Assesses regulatory intelligence to assist in the development of local, regional and global regulatory strategies.<br>• Evaluates the regulatory environment and contributes to providing internal advice throughout the product lifecycle (e.g., concept, development, manufacturing, marketing) to ensure product compliance.<br>• Anticipates regulatory obstacles and emerging issues throughout the product lifecycle and develops solutions.<br>• Identifies requirements and potential obstacles for market access and distribution (federal, provincial/territorial state, reimbursement, purchasing groups, HTA, etc.).<br>• Assists in the development of regulatory strategy and updates strategy based upon regulatory changes.<br>• Evaluates proposed products for regulatory classification and jurisdiction.<br>• Determines requirements (local, national, international) and options for regulatory submissions, approval pathways and compliance activities.<br>• Provides regulatory information and guidance for product development and planning throughout the product lifecycle to the regulatory unit and other units in the organization.<br>• Compares regulatory outcomes with initial product concepts and recommends changes or refinements based on initial regulatory outcomes.<br>• Negotiates with regulatory authorities throughout the product lifecycle.<br>• Identifies the need for new regulatory procedures and SOPs, and participates in development and implementation.<br>• Helps train stakeholders on current and new regulatory requirements to ensure organization-wide compliance.<br>• Assists other departments in the development of SOPs to ensure regulatory compliance.
**Product Development & Registration:** Knowledge of the research and development, preclinical and clinical steps, and related regulations in healthcare product development.
• Provides regulatory input and technical guidance on global regulatory requirements to product development teams.<br>• Advises stakeholders of regulatory requirements for quality, preclinical and clinical data to meet applicable regulations.<br>• Assesses the acceptability of quality, preclinical and clinical documentation for submission filing to comply with applicable regulations.<br>• Evaluates proposed preclinical, clinical and manufacturing changes for regulatory filing solutions and proposes plans/strategizes (if appropriate) for changes that do not require submissions.<br>• Provides knowledge and guidance on preapproval inspections, GCP inspections and clinical investigator relationships.<br>• Identifies, monitors and submits applicable reports (e.g., Serious Adverse Events) or notifications (e.g., changes in manufacturing) to regulatory authorities during the clinical research process.<br>• Provides regulatory information and guidance for proposed product claims/labeling.<br>• Ensures that the clinical and nonclinical data—in conjunction with regulatory strategy—are consistent with the regulatory requirements and support the proposed product claims.<br>• Prepares and submits electronic and paper regulatory submissions according to applicable regulatory requirements and guidelines.<br>• Monitors the progress of the regulatory authority review process through appropriate communication with the agency.<br>• Communicates and interacts with regulatory authorities before and during the development and review of a regulatory submission through appropriate communication tools.<br>• Works with cross functional teams for interactions with regulatory authorities including panel meetings and advisory committees.
**Postapproval/Postmarket:** Knowledge of requirements and processes to maintain a product on the market; reporting and surveillance.
• Develops and implements processes involved with maintaining annual licenses, registrations and listings.<br>• Reviews and approves advertising and promotion to ensure regulatory compliance.<br>• Assures postmarket regulatory requirements are met (e.g., required reports, supplemental submissions and other postmarketing commitments.)<br>• Submits notifiable changes and supplemental dossiers to the appropriate regulatory authorities to update product information and/or instructions for use to reflect current state of product knowledge.<br>• Participates in implementation of regulatory strategy and processes for handling recalls and communication to stakeholders (e.g., Dear Healthcare Professional letters, patient letters, distributor letters, and health authorities).<br>• Reports product safety issues to regulatory authorities as required, to comply with local, regional and global regulations.<br>• Provides required information (e.g., clinical data) in support of product reimbursement requests.<br>• Provides regulatory input and appropriate follow-up for inspections and audits.

Level II
**Scientific & Health Concepts:** Understanding and application of evolving basic and translational science, regulatory science and public health to drive new approaches to improve the development, review and oversight of healthcare products.
• Keeps abreast of and assesses the scientific and/or clinical advances that impact healthcare product development and regulation.<br>• Participates in stakeholder groups to help shape science-based regulatory decision making.
**Ethics:** Ability to integrate and demonstrate core values, integrity and accountability throughout the organization and externally.
• Demonstrates ethical behavior by ensuring integrity in personal and organizational practices; respects people and principles, including professional, ethical and people values, and serves as a role model for others.<br>• Contributes to building a respectful, diverse and inclusive workplace, where decisions and transactions are transparently and fairly.<br>• Holds one's self, one's employees and one's organization accountable for their actions.<br>• Abides by and upholds the laws and regulations of the authorities under which he or she operates and his or her organization's internal and external policies and directives.<br>• Takes all possible steps to prevent and resolve any real, apparent or potential conflicts of interest between one's official responsibilities and his or her private affairs.<br>• Raises and escalates, as appropriate, significant organizational ethics and compliance issues.
**Communication:** Ability to clearly convey or exchange information with stakeholders within and outside the organization.
• Clearly conveys or exchanges information with stakeholders within and outside the organization in an appropriate and timely manner.<br>• Prepares for and participates in meetings with internal and external stakeholders.<br>• Communicates with peers and supervisors and ensures alignment on issues, questions and goals.<br>• Escalates and effectively communicates issues to supervisor and other relevant staff.<br>• Aligns resources and discusses regulatory issues in cross-functional teams to ensure completion of project tasks.<br>• Prepares strategy/briefing documents for panel hearings and informational meetings.<br>• Communicates/refers external requests for information to the appropriate individual.
**Business Acumen:** Leverages systems and processes to successfully operate a regulatory function.
• Preserves confidentiality of product information as appropriate.<br>• Assures alignment of regulatory information management systems with other organizational systems.<br>• Frames issues with a thorough understanding of legislation, regulations, guidance, policy and directives.<br>• Identifies key resources and personnel for the project team—internal and external to his or her direct area of responsibility.<br>• Reviews change controls to determine the level of change and consequent submission requirements.<br>• Continually improves the quality of policies, programs and services provided.<br>• Manages and trains regulatory professionals.<br>• Creates work plans with appropriate staging of activities and with clearly defined milestones.<br>• Understands financial information used to make department/unit and organization-wide decisions and assists in the development and monitoring of department/unit budgets.
**Leadership:** Ability to direct and contribute to initiatives within the organization, with groups engaged in the development of good regulatory practice and policy, and within the regulatory profession.
• Seeks out diverse ideas, opinions, and insights and applies them in workplace.<br>• Connects and relates well with people who think and act differently than oneself.<br>• Embraces scrutiny and accepts feedback as opportunity to learn and improve.<br>• Chooses the best alternative to achieve desired outcome or effect, giving consideration to risks, tradeoffs, timing and available resources.<br>• Navigates the dynamics, alliances and competing requirements of the organization or business.<br>• Willingly accepts challenging assignments and new career opportunities that stretch and build capabilities.

LEVEL III

Level III professionals understand and translate regulatory, scientific, operational and business knowledge into effective implementation plans and strategy. This level represents the successful transition from technical and tactical regulatory expertise into a role that integrates technical knowledge, management and strategy. (Typical job titles at this level: director in private sector; senior/experienced reviewer, section manager in regulatory agencies)

Level III

Regulatory Frameworks & Strategy: Knowledge of regulatory frameworks and external environments, and the ability to apply these to regulatory solutions throughout the product lifecycle.

- Assesses links between global, societal and economic trends; stakeholder concerns and regulatory issues and requirements; and the implications for regulatory strategy.
- Develops and updates global, regional and multicountry regulatory strategy, and aligns regulatory strategies to organizational strategies.
- Provides guidance to integrate regulatory considerations into global product entry and exit strategy.
- Identifies regulatory pathways for initial product designs and provides input to internal stakeholders.
- Analyzes the adequacy of proposed regulatory pathways and strategy for initial product designs and recommends changes or refinements based on initial regulatory outcomes.
- Assesses all requirements and potential obstacles for market access and distribution (federal, provincial/territorial/state, reimbursement, purchasing groups, HTA, etc.) and develops solutions to address anticipated obstacles.
- Critically assesses the impact of changing regulations on preapproval and postapproval strategies and approaches and advises internal stakeholders on a course of action.
- Approves and executes changes to preapproval and postapproval strategies and approaches based on changing regulations.
- Provides in-depth understanding and ability to incorporate regulatory strategies to expedite development for products intended for serious or life-threatening medical conditions or that address unmet medical needs (e.g., orphan, conditional approval, breakthrough therapy).
- Negotiates with regulatory authorities on complex issues throughout the product lifecycle.
- Establishes working relationships and interfaces and with multiple government and non-government organizations having an impact on market access and distribution.
- Identifies the need for and manages the development and execution of new regulatory procedures and standard operating procedures (SOPs).
- Develops and manages programs that train stakeholders on current and new regulatory requirements to ensure organization-wide compliance.

Product Development & Registration: Knowledge of the research and development, preclinical and clinical steps, and related regulations in healthcare product development.

- Provides strategic input and technical guidance on global regulatory requirements to product development teams.
- Evaluates risks of product and clinical safety issues during clinical phases and recommends regulatory solutions.
- Evaluates proposed preclinical, clinical and manufacturing changes for regulatory filing solutions and proposes plans for changes that do not require submissions.
- Reviews and assesses proposals to regulatory authorities on regulatory paths and clinical plans.
- Monitors implementation of regulatory strategies relative to product and clinical safety issues identified during clinical phases.
- Provides knowledge and critical analysis of preapproval inspections, GCP inspections and clinical investigator relationships.
- Provides regulatory guidance on strategy for proposed product claims/labeling.
- Ensures clinical and nonclinical data are consistent with the regulatory requirements and support the proposed product claims.
- Manages electronic (eCTD) and paper registration development.
- Ensures policies and procedures are in place for appropriate internal review and approval of regulatory submissions.
- Leads key negotiations and interactions with regulatory authorities during all stages of the development and review process.
- Prepares cross functional teams for interactions with regulatory authorities including panel meetings/advisory committees.

Postapproval/Postmarket: Knowledge of requirements and processes to maintain a product on the market; reporting and surveillance.

- Ensures process is in place for review and approval of advertising and promotion to ensure regulatory compliance.
- Develops, implements and manages systems to track required reports, supplemental submissions and other postmarketing commitments.
- Reviews and approves required reports, supplemental submissions and other postmarketing commitments to maintain product registrations.
- Reviews and approves change controls to determine the level of change and consequent submission requirements.
- Develops, implements and manages appropriate SOPs and systems to track, manage and report and communicate product-associated event complaints, recalls, market withdrawals and vigilance reports.
- Adapts postmarket strategy based on consideration of factors such as HTA, reimbursement, group purchasing pressures, state/provincial/regional restrictions and other legislative/regulatory requirements.

Scientific & Health Concepts: Understanding and application of evolving basic and translational science, regulatory science and public health to drive new approaches to improve the development, review and oversight of healthcare products.

- Remains up-to-date on scientific and clinical advances that impact healthcare product development and assesses the relationship to regulation and regulatory issues.
- Actively engages with stakeholder groups to help shape science based regulatory decision making.

Level III

Ethics: Ability to integrate and demonstrate core values, integrity and accountability throughout the organization and externally.

- Abides by and upholds the laws and regulations of the authorities under which he or she operates and his or her organization's internal/external policies and directives.
- Holds one's self, one's employees and one's organization accountable for their actions.
- Takes all possible steps to prevent and resolve any real, apparent or potential conflicts of interest between one's official responsibilities and his or her private affairs.
- Raises and escalates, as appropriate, significant organizational ethics and compliance issues.
- Champions ethical behavior by ensuring integrity in personal and organizational practices; respect people and principles including professional, ethical and human values.
- Demonstrates and proactively advocates working together in a spirit of openness, honesty and transparency that encourages engagement, collaboration, respectful interactions and trust.

Communication: Ability to clearly convey or exchange information with stakeholders within and outside the organization.

- Clearly conveys or exchanges information with stakeholders within and outside the organization in an appropriate and timely manner.
- Delivers key messages effectively to a wide variety of audiences at all organizational levels.
- Organizes, prepares for and facilitates effective meetings with internal and external stakeholders.
- Communicates with peers and supervisors and ensures alignment on issues, questions and goals.
- Aligns resources and discusses regulatory issues in cross-functional teams to ensure completion of project tasks.

Business Acumen: Leverages systems and processes to successfully operate a regulatory function.

- Preserves confidentiality of product information as appropriate.
- Assures alignment of regulatory information management systems with other organizational systems.
- Leads integrated regulatory process and system improvement initiatives that will influence and build new capabilities for greater effectiveness and efficiencies.
- Ensures that corporate policies are adequate for compliance.
- Continually improves the quality of internal regulatory policies, programs and services.
- Evaluates regulatory risks of corporate policies.
- Frames issues with a thorough understanding of legislation, regulations, guidance, policy and directives.
- Performs regulatory due diligence and identifies risks and opportunities for executive management (such as M&A).
- Uses financial analysis to generate, evaluate and act on strategic options and opportunities to make business decisions.
- Integrates qualitative and quantitative information to draw accurate conclusions.
- Creates work plans with appropriate staging of activities and with clearly defined milestones.
- Identifies and connects talent needs with talent resources and recruits, retains, manages and develops regulatory professionals.

Leadership: Ability to direct and contribute to initiatives within the organization, with groups engaged in the development of good regulatory practice and policy, and within the regulatory profession.

- Articulates the organization's strategic vision in a manner that enables others to execute plans, tactics and actions.
- Continually identifies and informs appropriate individuals on emerging trends, opportunities and threats.
- Leverages a well-grounded knowledge of applicable laws, regulations and policies to develop and execute plans and programs.
- Ensures strategies, analyses and plans consider anticipated long-range requirements and are not just based on the current situation.
- Makes informed decisions based on business frameworks and tools and give consideration to risks, tradeoffs, timing and available resources.
- Proactively manages and monitors progress against desired outcomes including working with others to establish and adjust contingency plans, revising and adapting processes, communicating success and learning from mistakes.
- Demonstrates the ability to build agreement and acceptance through his or her ability to present a compelling case for ideas, negotiate persuasively, and address disagreements constructively.
- Continuously develops staff by making accurate assessments of individuals' capabilities and performance, and providing feedback, coaching, guidance and mentoring.
- Demonstrates sensitivity and understanding of cultural considerations when dealing with others.
- Embraces scrutiny and accepts feedback as opportunity to learn and improve.
- Willingly accepts challenging assignments and new career opportunities that stretch and build capabilities.

LEVEL IV

Level IV professionals take on the role of the strategic lead representing regulatory expertise while developing new approaches for business objectives. Strategic planning and interfacing throughout the product lifecycle—both within and outside the organization—are among the most important responsibilities at this level. These professionals must be able to navigate ambiguity, demonstrate agility, and those characteristics expected of executive-level individuals. They must possess and communicate a strong understanding of the requirements, opportunities, risks and alternatives for developing and maintaining products. (Typical job titles at this level: vice president, executive director, CEO in private sector; division director in regulatory agencies)

Level IV
**Regulatory Frameworks & Strategy:** Knowledge of regulatory frameworks and external environments, and the ability to apply these to regulatory solutions throughout the product lifecycle.
• Develops the organization's national, regional and global regulatory position(s) and strategy based upon assessment and synthesis of internal and external intelligence (opportunities/risks).<br>• Develops product positioning strategies for complex and/or critical products based upon current regulatory requirements and planned regulatory changes.<br>• Accesses and interprets environmental scans and other socioeconomic, scientific and regulatory intelligence to better understand and contribute product positioning, competition, opportunities and to drive regulatory strategy.<br>• Integrates regulatory considerations into the organization's global product entry and exit strategy.<br>• Identifies issues early in the development or research phase that could impact regulatory strategy, submissions and/or product launches for complex and/or critical products.<br>• Manages negotiations with regulatory authorities on complex issues throughout the product lifecycle.<br>• Influences changing regulations and guidance.<br>• Interfaces with and establishes working relationships with multiple government and non-government organizations impacting market access and distribution.<br>• Leads efforts to incorporate regulatory strategies to expedite development for products intended for serious or life-threatening medical conditions or that address unmet medical needs (e.g., orphan, conditional approval, breakthrough therapy).<br>• Leads negotiations with regulatory and other health authorities on complex issues throughout the product lifecycle.<br>• Leads the development and execution of good regulatory practice and policy.
**Product Development & Registration:** Knowledge of the research and development, preclinical and clinical steps, and related regulations in healthcare product development.
• Provides strategic input on regulatory requirements to R&D and clinical leads for complex and/or critical products.<br>• Leads the regulatory team's engagement in evaluation of risk and safety issues for complex and/or critical products and recommends regulatory solutions during preapproval/clinical phases.<br>• Participates in risk-based decisions on compassionate use/special access approvals based upon patient needs and risk assessment.<br>• Approves regulatory filing strategies for complex and/or critical products based upon proposed preclinical, clinical and manufacturing changes.<br>• Reviews and approves publicly disseminated information on product submission approval status.
**Postapproval/Postmarket:** Knowledge of requirements and processes to maintain a product on the market; reporting and surveillance.
• Reviews and approves enforcement action/responses.<br>• Integrates changes in postmarket strategy based upon consideration of factors such as HTA, reimbursement, group purchasing pressures, state/provincial/regional restrictions and other legislative/regulatory requirements to organization's business strategies.<br>• Leads and represents the regulatory team in product associated events, recalls and product withdrawals.
**Scientific & Health Concepts:** Understanding and application of evolving basic and translational science, regulatory science and public health to drive new approaches to improve the development, review and oversight of healthcare products.
• Serves as a thought leader in the understanding and application of evolving basic and translational science, regulatory science and public health to develop new approaches to improve the development, review and oversight of healthcare products.<br>• Identifies and proactively responds to scientific and/or clinical advances that impact healthcare product development and regulation.

Level IV

Ethics: Ability to integrate and demonstrate core values, integrity and accountability throughout the organization and externally.

- Applies the Code of Ethics for the Regulatory Profession and develops an organizational code for regulatory and related staff.
- Educates internal stakeholders on the relevance and importance of ethics to the organization.
- Champions ethical behavior by ensuring integrity in personal and organizational practices, respect for people and principles, including professional, ethical and human values.
- Holds one's self, one's employees and one's organization accountable for their actions.
- Abides by and upholds the laws and regulations of the authorities under which he or she operates and his or her organization's internal/external policies and directives.
- Demonstrates and proactively advocates working together in a spirit of openness, honesty and transparency that encourages engagement, collaboration, respectful interactions and trust.
- Takes all possible steps to prevent and resolve any real, apparent or potential conflicts of interest between one's official responsibilities and one's private affairs.
- Raises and escalates, as appropriate, significant organizational ethics and compliance issues.

Communication: Ability to clearly convey or exchange information with stakeholders within and outside the organization.

- Communicates the organization's regulatory positon to business partners.
- Communicates updates to staff to gain alignment.
- Develops and implements effective communication and engagement strategies with partners.
- Communicates with knowledge, consistency, and clarity to maintain integrity and impact of the message.

Business Acumen: Leverages systems and processes to successfully operate a regulatory function.

- Assesses the data/metrics/performance etc. generated for continuous improvement opportunities within the organization and leverages such information to achieve regulatory objectives.
- Creates a culture of good information practices, protection/safeguarding of information.
- Preserves confidentiality of product information as appropriate.
- Provides strategic guidance for resource and development planning.
- Understands and utilizes financial information to contribute to organizational business decisions and to make regulatory business unit decisions.
- Develops strategies for resolving complex issues with potential for significant regulatory impact.
- Provides recommendations to decision makers on regulatory strategies and options on new products or claims that balance business needs with regulatory oversight.
- Oversees regulatory aspects of business relationships to ensure compliance and protect corporate interests.
- Creates or modifies operational infrastructures (e.g., processes, systems, structures, roles, metrics) to support strategic objectives and for driving sustainable results.
- Negotiates regulatory and scientific issues with management.

Leadership: Ability to direct and contribute to initiatives within the organization, with groups engaged in the development of good regulatory practice and policy, and within the regulatory profession.

- Wins support and buy-in from sponsors, partners and stakeholders by effectively satisfying stakeholder interests and concerns while advocating a clear direction forward; and coaches others to do the same.
- Models, encourages and creates the conditions for an inclusive and respectful work environment. Acts as a catalyst and change agent for creating an inclusive and respectful work environment.
- Models humility by readily taking responsibility for errors, acknowledging opportunities for improvement, pursuing new ideas and perspectives and applying learning.
- Actively engages in talent management practices (selection, promotion, development and engagement) to cultivate a workforce that is well aligned with current and emerging talent needs.
- Builds and sustains partnership across organizational boundaries and functions as well as outside the organization to achieve common goals and outcomes.
- Leads thorough analysis of situations with appropriate attention to details and the big picture including consideration of impact at multiple levels of the system.
- Makes timely and effective decisions, balancing the need for more information or analysis with the need to be decisive.
- Makes tough or unpopular decisions where mission outcomes supersede the interests/concerns of individuals, constituencies or current situation.
- Factors cultural considerations and impact into decision making.
- Actively leads and engages in policy development, implementation and communication by framing emerging issues and contributing expertise in support of the organization's vision, strategy, priorities and obligations.
- Ensures knowledge and lessons learned are shared across organizational boundaries.
